# Antenatal corticosteroid therapy for foetal maturation in women with eclampsia and severe pre-eclampsia in a rural hospital in Western Tanzania

**DOI:** 10.1186/s12884-016-1023-8

**Published:** 2016-08-19

**Authors:** R. Mooij, I. H. Mwampagatwa, J. van Dillen, J. Stekelenburg

**Affiliations:** 1 Ndala Hospital, 15 Ndala, Tabora, Tanzania; 2 Department of Obstetrics and Gynaecology, Maastricht University Medical Centre, P. Debyelaan 25, 6229 HX Maastricht, The Netherlands; 3 College of Health Sciences, University of Dodoma, 395, Dodoma, Tanzania; 4 Department of Obstetrics and Gynaecology, Radboud University Medical Centre, Geert Grooteplein-Zuid 10, 6525 GA Nijmegen, The Netherlands; 5 Department of Obstetrics and Gynaecology, Leeuwarden Medical Centre, Henri Dunantweg 2, 8934 AD Leeuwarden, The Netherlands; 6 Department of Health Sciences, Global Health, University Medical Centre Groningen/University of Groningen, PO Box 196, 9700 AD Groningen, The Netherlands

**Keywords:** Low-income countries, Preterm birth, Glucocorticoids, Antenatal corticosteroid therapy, Tanzania

## Abstract

**Background:**

Preterm birth is a major cause of neonatal mortality, especially in low and middle income countries. Antenatal corticosteroid therapy for foetal maturation could have a significant impact and therefore is often referred to as an important strategy to reduce neonatal mortality. A recently conducted large multinational trial showed that antenatal corticosteroids can have adverse effects in low income countries, but this is likely to depend on the specific setting. In our hospital preterm birth is only recognized in patients with severe maternal disease, due to physician-initiated delivery. Spontaneous preterm births are rarely seen in the hospital and often take place in the community or while on the road to a health facility.

**Objective:**

To investigate the effects of antenatal corticosteroid therapy in a rural hospital in Tanzania.

**Methods:**

A secondary analysis of a retrospective medical records study of women with severe pre-eclampsia and eclampsia performed in Ndala Hospital between July 2011 and December 2012. We used data on gestational age, birth weight, Apgar score, time between admission and birth, use of corticosteroids and maternal and foetal survival. Ethical clearance was obtained from the directorate of research and publications of the University of Dodoma (ref. UDOM/DRP/346).

**Results:**

Thirty-six women with forty live foetuses were analysed. Twelve women (13 neonates) were given corticosteroids and could be compared to 24 women (27 neonates) who did not get corticosteroids. The incidence of fresh stillbirths (antenatal death) was 20 %. The 13 neonates who received corticosteroids had significantly smaller birth weight, longer interval between admission and delivery and poorer outcomes (stillbirth and neonatal death). An analysis of 24 neonates with a birth weight between 1.5 and 2.5 kg showed a trend toward better outcome in neonates who did not receive antenatal corticosteroid therapy.

**Conclusion:**

Small retrospective studies as these have a low level of evidence, but this study helped to gain more knowledge of local conditions affecting the effectiveness of antenatal corticosteroid therapy in our setting of a small rural hospital. Reliability of estimating gestational age, epidemiology of preterm birth, exposure to infections, foetal monitoring and quality of neonatal care are likely to influence the effect of antenatal corticosteroid therapy. Further larger prospective studies should be conducted to determine the exact preconditions of antenatal corticosteroid therapy in low-income countries. Until that time, the WHO precautions seem reasonable and audits and small observational studies like ours can help in assessing whether a specific hospital is suited for antenatal corticosteroid therapy.

## Background

Almost 10 % of births worldwide are preterm and the incidence increases to 20 % in parts of Africa [1]. Causing one million neonatal deaths each year, most of which occur in low- and middle-income countries, preterm birth is a major cause of neonatal mortality [2]. The rate of preterm birth is not expected to fall and might even increase, partly because of lack of preventive measures and partly because of physician-initiated deliveries for various conditions [3]. Antenatal corticosteroid therapy (ACT) for foetal maturation could have a significant impact on neonatal survival [4, 5].

Corticosteroids trigger the maturational process leading to the release of surfactant into the alveoli of the foetal lung, preventing respiratory distress syndrome [6]. ACT for foetal maturation has been undisputed since the publication by Liggins and Howie in 1972 [7], although long-term health effects have been less well studied [8]. In the most recent Cochrane systematic review of 18 trials the effect there was a 34 % reduction of respiratory distress syndrome, a 46 % reduction of intraventricular haemorrhage and a 31 % reduction in neonatal mortality [9]. The use of ACT is incorporated in many guidelines [9–11]. Recently a study has shown positive effects for late preterm birth as well [12].

A review in middle-income countries (Brazil, Jordan, Tunesia and South Africa) showed a mortality reduction of 53 % [13]. The authors remark that the effect in low-income countries (LIC) might be even larger due to lack of neonatal health care facilities and limited access to expensive interventions such as surfactant therapy. Hence, ACT is often referred to as an important strategy to reduce neonatal mortality in LIC [5, 14–20]. In Tanzania ACT has been listed as a cost effective measure in the 2008 national road map strategic plan to reduce newborn and child deaths [21, 22].

However, some doubts were voiced about ACT in LIC [23], which were reinforced when in 2015 a LIC-ACT trial was published in The Lancet by Althabe et al. [24]. This large trial investigated ACT implementation versus regular care in 100,000 women in six countries (Argentina, Guatalamala, India, Kenya, Pakistan, Zimbabwe). This population based study did not show a positive effect in the preterm infants group, even though nearly half of them received ACT. In this study, an increase in overall perinatal and neonatal mortality in the whole group was shown [24, 25], probably due to deleterious effects of overtreatment by ACT in patients who were not preterm (84 %). Also there was an increase in suspected maternal infection and an increased maternal mortality ratio. This trial was conducted mostly at community level with only 13 % of women identified for ACT in a hospital.

In response to these findings, the World Health Organization (WHO) has recommended the following conditions to be met before ACT administration for women at risk for preterm birth between 24 and 34 weeks of gestation: Gestational age (GA) can be accurately assessed, preterm birth is imminent, there is no evidence of infection and adequate childbirth care and care for the preterm neonate are available [26, 27].

We were interested to examine the situation in our local hospital. To assess the effects of ACT in a hospital in a low-resource setting in rural Africa, we analysed data from a subset of women included in a previous retrospective study conducted in women with severe pre-eclampsia and eclampsia. Even though spontaneous preterm labour is an indication for ACT in the national and hospital protocols, in our hospital hypertensive disorders in pregnancy are the most important indications for ACT.

## Methods

### Setting

This study was done at Ndala Hospital, a private Catholic hospital, situated in the Tabora region, in a rural part of Western Tanzania. It serves a catchment area of approximately 200,000 people. Annually, approximately 2,200 women give birth in the hospital. Comprehensive emergency obstetric care is available. There are virtually no possibilities for urgent referral to the regional hospital. Monitoring of foetal wellbeing is done by a foetal heart doppler once daily with admitted patients and more regularly during labour (4 hourly during 1st stage of labour, every 15 min during 2nd stage). Cardiotocography (CTG) is not available. Obstetric ultrasound is available, but is used for specific indications only and not routinely for estimation of GA or foetal biometry. Because of late booking 1st and early 2nd trimester ultrasound is rarely done. GA is calculated using maternal history of last menstrual period or, if unknown, by measuring fundal height. In case there is a discrepancy between maternal history and fundal height, the clinician decides which GA is most likely, sometimes helped by information from earlier visits to the antenatal clinic. Ultrasound measurement of foetal biometry is not used in these patients because of limited resources and insufficiently trained healthcare workers. Neonatal resuscitation is done by mask and balloon only; there are no possibilities for assisted ventilation. Premature neonates are admitted with their mothers in the premature room for frequent cup feeding and Kangaroo Mother Care.

### Participants

**Fig. 1:**
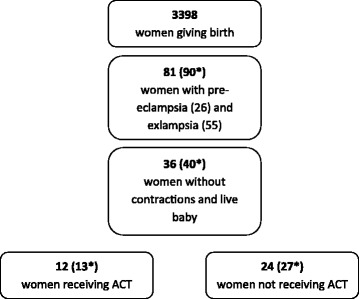
Patient selection (number of foetuses*)

This is a secondary analysis of a retrospective medical records study of women with severe pre-eclampsia and eclampsia performed in Ndala Hospital between July 2011 and December 2012 [28]. During this period 3398 women gave birth in the hospital. Eighty-one patients (90 neonates) were included in the original study (Fig. 1). In the present study, we included a subset of neonates from 36 women without contractions (40 live foetuses). The patients were identified by the first author (RM) or one of the attending doctors. Medical records were searched immediately following discharge or death and a standard case record form was filled in by the discharging doctor and cross-checked. In case of discrepancies or missing data, the medical records were checked again. For this analysis about the effect of ACT, we used data on GA, birth weight, Apgar score, time between admission and birth, use of corticosteroids and maternal and foetal survival. We performed a subgroup analysis of neonates with a birth weight between 1.5 and 2.5 kg, to try to compensate for indication bias.

The use of ACT was considered in cases of severe pre-eclampsia in mothers without contractions and suspected prematurity (GA suspected < 34 weeks or < 2.5 kg) with a live baby. The decision to start ACT was based on the estimated GA, maternal condition and the preferences of the patient or her relatives. In case of eclampsia, although the hospital protocol advises prompt induction of labour after stabilizing the maternal condition, this was sometimes delayed and ACT was given outside of the protocol.. Overtreatment was defined as ACT given when there was no low birth weight, which means that the weight was over 2.5 kg [29]. Dexamethasone was given (8 mg intra-muscular (IM)) and repeated once after 24 h. The course was considered completed 48 h after the first dose.

### Statistical analysis

Data management was done using Microsoft Excel®, statistical analysis was done with Epi Info®. P-values were calculated with Fisher-Exact test, *T*-test and Mann-Whitney /Wilcoxon test, whether appropriate.

## Results

Thirty-six women who were not in labour on admission carried 40 live foetuses. Twelve women (13 neonates) were given ACT and could be compared to 24 women (27 neonates) who did not get ACT. Sixty-seven percent of women (24/36) had eclampsia, while 33 % (12/36) had severe pre-eclampsia and this was similar in women who had received ACT and who had not. All but one woman received magnesiumsulphate. Of all these women all data could be retrieved. One woman received an incomplete dose of ACT: she gave birth to a baby of 1.3 kg, which died after delivery. Data of this patient were analysed in the group of women receiving corticosteroids (intention to treat analysis).

The overall neonatal survival was 68 % (27/40), with a mean birth weight of 2.2 kg. The incidence of fresh stillbirths (antenatal death) was 20 %. The thirteen neonates who received ACT had significantly smaller birth weight, longer interval between admission and delivery and poorer outcomes (see Table 1). Overtreatment (ACT given to foetuses >2.5 kg) occurred in 38 % of ACT courses. One maternal death occurred (bleeding complication of Caesarean section) in a woman who had not received ACT. Induction with misoprostol was done in twenty-two women (25/27 foetuses, 93 %) who did not receive ACT and in eight women (8/13 foetuses, 62 %) who received ACT.

**Table 1:** Characteristics of 40 neonates of women not in labour

Number of neonates (women^a^)	Received ACT: 13 (1 incomplete) (12^a^)	Not received ACT: 27 (24^a^)	*P*-value
Pre-eclampsia/eclampsia			
Eclampsia	9 (8^a^) (69 %)	17 (16^a^) (63 %)	1 (Fisher)
Pre-eclampsia	4 (31 %)	10 (8^a^) (37 %)	
Maternal outcome			
Mother died	0 (0 %)	1 (3.7 %)	1 (Fisher)
Mother survived	13 (11^a^) (100 %)	26 (23^a^) (96 %)	
Multiple gestation			
Twins	2 (1^a^) (15 %)	6 (3^a^) (22 %)	1 (Fisher)
Singleton	11 (85 %)	21 (78 %)	
Median self-reported gestational age (months, interquartile-range)	7 (7–8)	8 (8–9)	<0.01 (MW/Wilcoxon)
Average weight (range)	1.8 kg (0.8–3.2 kg)	2.4 kg (1.5–4 kg)	0,03 (*T*-test)
Median between admission-delivery (hours, interquartile-range)	147 (72–200)	42 (17–72)	<0.01 (MW/Wilcoxon)
Perinatal outcome			
Alive child at discharge	5 (38 %)	22 (81 %)	<0.01 (Fisher)
Perinatal death	8 (62 %)	4 (19 %)	
Intrauterine death	5 (38 %)	3 (11 %)	
Labour			
Spontaneous labour & delivery	3 (23 %)	2 (7 %)	0.3 (Fisher)
Primary SC	2 (1^a^) (15 %)	0 (0 %)	
Induction, SVD	6	21 (18^a^)	
Failed induction, secondary SC	1		
Successful induction, secondary SC when in labour	1	3	
Induction, ventouse delivery		1	

^a^twins: number of women, calculations are with foetuses

All four neonates with a birth weight < 1.5 kg died before or during birth (all received corticosteroids). Of the twelve children above 2.5 kg ten survived (83 %). None of the two children that died (one antenatal, one postnatal) had suspected respiratory distress syndrome (RDS). Of the neonates who received ACT, 4 (4/13 = 31 %) had a birth weight < 1.5 kg and 1 (1/13 = 8 %) a birth weight above 2.5 kg.

This subgroup of neonates with a birth weight between 1.5 and 2.5 kg consists of eight neonates who received ACT and sixteen who did not. In both groups the birth weights and reported GA were not significantly different. In the group who did not receive ACT, the admission to delivery interval was significantly shorter and neonatal outcome better (not significant) (Table 2). Thirty-eight percent (5/13) of women receiving ACT carried foetuses of less than 1.5 kg or more than 2.5 kg.

**Table 2:** characteristics of 24 neonates of women not in labour between 1.5 and 2.5 kg

Number of neonates (women^a^)	Received ACT: 8 (7^a^)	Not received ACT: 16 (13^a^)	*P*-value
Median self-reported gestational age (months, interquartile-range)	8 (7–8)	8 (8–9)	0.31 (*T*-test)
Average weight (range)	2.1 kg (1.5–2.4 kg)	1.9 kg (1.5–2.5 kg)	0.50 (MW/Wilcoxon)
Median between admission-delivery (hours, interquartile-range)	144 (96–250)	22 (16–76)	<0.01 (MW/Wilcoxon)
Perinatal outcome			
Alive child at discharge	4 (50 %)	13 (81 %)	0.17 (Fisher)
Perinatal death	4 (50 %)	3 (19 %)	
Intra-uterine death	2 (25 %)	1 (6,3 %)	

^a^twins: number of women, calculations are with foetuses

## Discussion

In our small group of 40 neonates in women with severe hypertensive disorder no benefit of ACT could be demonstrated. An analysis of 24 neonates with a birth weight between 1.5 and 2.5 kg showed a trend toward better outcome in neonates who did not receive ACT, however the results of this small retrospective study must be regarded with caution.

Overtreatment was defined as ACT given when the birth weight was over 2.5 kg. This is the 50th weight percentile at 35 weeks of GA. A small positive effect for late preterm birth has been established recently, but has not been incorporated in guidelines for LIC [9, 12, 30]. The cut-off birthweight in the LIC-ACT trial was calculated as 2000–2500 g depending on the country [24].

### Study limitations

Our study has several limitations.

Firstly, because of the retrospective setup, both groups had different characteristics. There is risk for indication bias as the reasons for starting ACT or initiating prompt delivery were not well described. For example, the decision to withhold ACT could have been based upon the estimation that either the baby would be too small or too large to benefit from ACT. The hospital protocol advises direct delivery without ACT in case of eclampsia, yet two-thirds of women had eclampsia and were treated outside of the protocol. This is because when the maternal condition improved after initial treatment with magnesiumsulphate the mother and relatives often refused preterm delivery or needed time to consider this, and ACT was given in the mean time.

Secondly, since GA (and estimated weight) was never exactly known, the inclusion for ACT was not done according to the correct GA (26–34 weeks), which can have led to underestimation of the positive effect of foetal maturation in case of small for gestational age infants. Our results underline the fact that medical decision making is difficult without known GA and show the advantage of early booking ultrasound to determine the exact GA. However in many LIC this is not realistic and in clinical practice, as well as studies, the use of proxies and clinical judgement is required [24].

We have tried to correct for both these limitations in Table 2, analyzing subgroups with comparable birth-weight (as a proxy for GA), however this analysis could have included small for gestational age (growth restricted) newborns as well and although the proportion of women with severe disease (eclampsia) is the same in both groups, the median self-reported GA and birth weight are slightly lower (non-significant) in the group that received ACT. The groups were too small to perform regression analysis for this confounder and we could not exclude other possible reasons for bias.

A third limitation is the setting: the study was not done in a general population of women with clinical signs of preterm birth, but done in women with indication for induction of labour because of severe pre-eclampsia and eclampsia. However, this is the clinical practice in our setting in which hypertensive disorder in pregnancy is the only circumstance in which ACT is given. In the study period there were no women without pre-eclampsia or eclampsia who received ACT, either because they did not arrive in time in the hospital (born before arrival or arrived in second stage of labour) or were not adequately identified on admission [31]. This limitation means the results cannot be generalized to other settings, but represent daily practice in our hospital.

Lastly, the dexamethasone schedule of 2 times 8 mg dexamethasone is a lower dose than the advised dosage of 4 times 6 mg IM in 48 h and might have been less effective.

Several authors have given explanations for increased risk and lower effectiveness of ACT in low-income countries [3, 19, 23, 25, 32]. We want to discuss several aspects of using ACT in low resource settings, using the findings of our study.

### Causes of inappropriate administration of ACT

- Uncertain GA: in a setting of late booking without availability of (early) ultrasound it is difficult to identify women who can benefit from ACT, since it is difficult to estimate foetal body weight and not possible to identify intra-uterine growth restriction. In our study there was a considerable overtreatment of 38 % of women who were not eligible to receive ACT. In the LIC-ACT trial of the 13 % of women receiving ACT, only 16 % delivered a preterm infant (in both studies using birth weight as a proxy for GA). The difference in overtreatment can be explained because in our group all women receiving ACT delivered shortly after (because of induced preterm birth), but the GA was uncertain. In the LIC-ACT trial, not only the GA was uncertain, but also if preterm delivery was really imminent. The elevated risk in overall newborn mortality, from 23.9 to 27.4 per 1000 and even more significant in African sites of the study, has been attributed mainly to the inappropriate administration of ACT to mothers who gave birth to neonates who were not premature [32–34]. Although positive effects have been shown in the late-preterm period, these are much smaller [12]. Not only will this dilute any effects of ACT, but women and babies are also exposed to the risk of corticosteroids without the benefits.
- Difficulty in identifying women with imminent preterm birth: although preterm labour is an important indication for ACT in high income settings, in many hospitals in low resource settings ACT is never used for this. Few women present with preterm labour and even if they do, often prematurity is not suspected since GA is not known. When preterm labour is suspected, arrival in the hospital can be too late to start tocolytics. There might be lack of awareness by healthcare workers and mothers. After health-provider training preterm labour was the most common indication (77 %) for ACT in the LIC-ACT trial and 70 % of women completed the course [24]. Ultimately more than half of women delivered at term, indicating that it is difficult to diagnose imminent preterm birth [35].
  Preterm rupture of membranes before 32 weeks GA is also a recognized indication for ACT [9, 36, 37]. These patients as well often do not seek medical attention in the setting of our study area and in the few cases that are admitted in our hospital, ACT is not given because of the fear of sepsis.
  In our hospital ACT is only given to women with severe maternal disease requiring termination of pregnancy, almost always severe hypertensive disorder. Although in high-income countries evidence of the benefits for this treatment regime is clear [9], a different analysis has to be made in LIC, since the risks are different. ACT requires postponing delivery for 48 h, while women with severe diseases have a serious risk of severe maternal morbidity or even maternal mortality and cannot always be monitored and treated adequately. Many women with severe hypertensive disorder in a low-resource setting already report late at health institutions as a result of delay caused by late recognition of dangers signs, challenges in the decision-making process to go to the hospital and/or transport problems [28].

### Increased risk of corticosteroids

- Risk of infections: corticosteroids increase susceptibility to infection and decrease immune function [3]. In LIC the infectious disease burden is higher and the level of antisepsis lower. Sepsis is a main cause of foetal and maternal mortality, so the effect of administering ACT is potentially dangerous. Corticosteroids are contra-indicated in patients with chronic infections [36]. HIV, malaria and tuberculosis (TB) are more common in LIC. The first can easily be excluded before administering ACT, but Tanzania has a high burden of tuberculosis with a prevalence of 176/100,000 in 2012 [38], with many patients being asymptomatic and unaware of their illness. In 2012, 138 patients were registered at the TB clinic and 2798 at the HIV Care and Treatment clinic of our hospital [39].

### Limited possibility for adequate preterm birth care and postnatal care

- Diagnosing and monitoring foetal and maternal condition: ACT is contra-indicated if there are signs of chorio-amnionitis [36]. Our study setting is characterised by an understaffed and overburdened labour ward, with limited possibilities of clinical monitoring like temperature and no CTG or laboratory markers such as C-reactive protein. This leads to inadequate identification of infection. Aside from infection, the foetus is also at a higher risk of intra-uterine death because of the mothers condition (e.g., insufficient placental function in eclampsia) or birth asphyxia. Without the possibility of foetal monitoring we found a high antenatal and perinatal mortality of 20 %. Keeping the foetus in this dangerous environment without the ability to check the foetal condition can lead to undetected foetal distress and eventually death.
- □ Neonatal care: causes of neonatal mortality of preterm infants in resource-limited settings are hypothermia, hypoglycaemia, birth asphyxia, infection and respiratory issues. To address only the latter cause when the others cannot be well managed has been called useless [40].

Our small observational study supports the recent findings that ACT is not always beneficial in LIC. Because of its heterogeneity the LIC-ACT trial is not applicable to all hospitals in LIC. Our study is limited by bias and small numbers, however it provides insight in the practice in a rural hospital. The WHO has recommended accurate assessment of GA, prediction of imminent preterm birth, evidence of no infection and adequate childbirth care and care for the preterm neonate to be present before ACT is implemented [26, 27]. Our data support these general recommendations. Further larger prospective studies can determine the exact preconditions of ACT in LIC. Until that time, audits and small observational studies like ours can help in assessing whether a specific hospital is suited for ACT.

## Conclusion

In forty neonates born prematurely due to mothers with severe pre-eclampsia and eclampsia no benefit of ACT could be demonstrated. An analysis of 24 neonates with a birth weight between 1.5 and 2.5 kg showed a trend toward better outcome in neonates who did not receive ACT. These results are in line with recent findings that ACT is not always beneficial. Small retrospective studies as these have a low level of evidence, but this study helped to gain more knowledge of local conditions affecting the effectiveness of ACT in our setting of a small rural hospital. Reliability of GA estimation, epidemiology of preterm birth, exposure to infections, foetal monitoring and quality of neonatal care are likely to influence the effect of ACT, but it is unclear what exact preconditions are required for ACT to be effective and safe. Further larger prospective studies should be conducted to determine the exact preconditions of ACT in LIC. Until that time, the WHO precautions seem reasonable and audits and small observational studies like ours can help in assessing whether a specific hospital is suited for ACT.

## Abbrevations

ACT: Antenatal corticosteroid therapy
CS: Caesarean section
CTG: Cardiotocography
GA: Gestational age
IM: Intra-muscular
LIC: Low-income countries
SVD: Spontaneous vaginal delivery
TB: Tuberculosis
WHO: World Health Organization
